# E26 Transformation-Specific-1 (ETS1) and WDFY Family Member 4 (WDFY4) Polymorphisms in Chinese Patients with Rheumatoid Arthritis

**DOI:** 10.3390/ijms15022712

**Published:** 2014-02-17

**Authors:** Yiqun Zhang, Lin Bo, Hui Zhang, Chao Zhuang, Ruiping Liu

**Affiliations:** 1 Department of Ophthalmology, Affiliated Hospital of Suzhou University, Changzhou No.4 People’s Hospital, Changzhou 213001, Jiangsu, China; E-Mail: yiqunzhang113@gmail.com; 2 Department of Rheumatology, the Second Affiliated Hospital of Soochow University, Suzhou 215002, Jiangsu, China; E-Mail: bolin20070101@163.com; 3 Department of Orthopedics, Affiliated Hospital of Nanjing Medical University, Changzhou Second People’s Hospital, Changzhou 213003, Jiangsu, China; E-Mails: zjzh1987@126.com (H.Z.); zhuangchaochina@gmail.com (C.Z.); 4 Central Laboratory, Affiliated Hospital of Nanjing Medical University, Changzhou Second People’s Hospital, Changzhou 213003, Jiangsu, China

**Keywords:** ETS1, WDFY4, polymorphisms, rheumatoid arthritis, molecular epidemiology

## Abstract

E26 transformation-specific-1 (ETS1) and WDFY family member 4 (WDFY4) are closely related with systemic lupus erythematosus. We hypothesized that *ETS1* and *WDFY4* polymorphisms may contribute to rheumatoid arthritis (RA) susceptibility. We studied *ETS1* rs1128334 G/A and *WDFY4* rs7097397 A/G gene polymorphisms in 329 patients with RA and 697 controls in a Chinese population. Genotyping was done using matrix-assisted laser desorption/ionization time-of-flight mass spectrometry. When the *WDFY4* rs7097397 AA homozygote genotype was used as the reference group, the AG genotype was associated with a significantly increased risk for RA. In the dominant model, when the *WDFY4* rs7097397 AA homozygote genotype was used as the reference group, the AG/GG genotypes were associated with a significant increased susceptibility to RA. In stratification analyses, a significantly increased risk for RA associated with the *WDFY4* rs7097397 AG genotype was evident among female patients, younger patients, C-reactive protein (CRP) negative patients and both anti-cyclic citrullinated peptide antibody (ACPA) positive patients and negative patients compared with the *WDFY4* rs7097397 AA genotype. These findings suggested that *WDFY4* rs7097397 A/G may be associated with the risk of RA, especially among younger, female patients, CRP-negative patients and both ACPA positive and negative patients. However, our results were obtained from a moderate-sized sample, and therefore this is a preliminary conclusion. To confirm these findings, validation by a larger study from a more diverse ethnic population is needed.

## 1. Introduction

Rheumatoid arthritis (RA) is one type of systemic autoimmune diseases due to a failure of immune self-tolerance. RA is characterized by synovial inflammation and hyperplasia, autoantibody production protein antibody, cartilage and bone destruction and systemic features [1]. RA is a complex disease with genetic and environmental predisposing factors. The genetic variants may contribute 50%–60% of the etiology of RA [2]. The highly polymorphic HLA region is a major contributor and accounts for approximately one-third of genetic risk of RA [3]. However, other additional risk alleles of RA remain to be identified [4].

RA and systemic lupus erythematosus (SLE) are autoimmune rheumatic diseases thought to have a substantial genetic contribution [5]. Recent genome-wide association studies in SLE have identified several novel associated locus including E26 transformation-specific-1 (ETS1) rs1128334 G/A and WDFY family member 4 (WDFY4) rs7097397 A/G polymorphisms, which have not been investigated in RA [6].

The ETS1 transcription factor is a member of the helix-turn-helix family [7]. ETS1 is required for angiogenesis and cell apoptosis [8]. In the synovial membrane of the joint in active RA patients, ETS1 is produced by endothelial cells and new blood vessels under pathological conditions [9,10]. ETS1 is present in T cells, B cells and natural killer cells [11,12]. A very high level of interleukin-10, an anti-inflammatory cytokine, has been observed in ETS1 deficient type 1 T helper cells [13]. In a recent investigation, ETS1 levels were strongly affected miR-146a promoter activity *in vitro*; and the knockdown of ETS1 directly impaired the induction of miR-146a [14]. High miR-146a expression levels were correlated with active disease in RA patients [15,16].

WDFY4 is predominantly expressed in the immune tissues. The function of WDFY4 is not well known; rs7097397 in *WDFY4* changes an arginine residue to glutamine (R1816Q) [6].

*ETS1* rs1128334 G/A and *WDFY4* rs7097397 A/G polymorphisms were distinctly associated with SLE [6]. However, further investigations between *ETS1* rs1128334 G/A and *WDFY4* rs7097397 A/G polymorphisms and RA risk were not conducted. We therefore undertook genotyping in a hospital-based case–control study in a cohort of 329 patients with RA and 697 controls in a Chinese population.

## 2. Results

### 2.1. Characteristics of the Study Population

Among 329 patients and 697 controls who provided DNA samples, genotyping for the *ETS1* rs1128334 G/A polymorphism was successful in 319 (97.0%) patients and 673 (96.6%) controls; genotyping for the *WDFY4* rs7097397 A/G polymorphism was successful in 321 (97.6%) patients and 691 (99.1%) controls. The demographic and clinical characteristics of all subjects are summarized in Table 1. Subjects were adequately matched for age and sex (*p* = 0.829 and 0.190, respectively). The genotype distributions of *ETS1* rs1128334 G/A and *WDFY4* rs7097397 A/G in all subjects are illustrated in Table 2. The observed genotype frequencies for the polymorphism in controls were in HWE for *ETS1* rs1128334 G/A (*p* = 0.570) and *WDFY4* rs7097397 A/G (*p* = 0.116).

### 2.2. Associations between *ETS1* rs1128334 G/A and *WDFY4* rs7097397 A/G Polymorphism and the Risk of RA

The genotype frequencies of the *ETS1* rs1128334 G/A polymorphism were 42.3% (GG), 44.5% (GA) and 13.2% (AA) in RA patients, and 43.2% (GG), 44.3% (GA) and 12.5% (AA) in controls (*p* = 0.939) (Table 2). Logistic regression analyses revealed that *ETS1* rs1128334 G/A polymorphisms were not associated with the risk of RA (Table 2).

The genotype frequencies of the *WDFY4* rs7097397 A/G polymorphism were 38.0% (AA), 49.8% (AG) and 12.1% (GG) in RA patients, and 46.9% (AA), 41.2% (AG) and 11.9% (GG) in controls (*p* = 0.022) (Table 2).

When the WDFY4 rs7097397 AA homozygote genotype was used as the reference group, the AG genotype was associated with a significantly increased risk for RA (OR = 1.49, 95% CI = 1.12–1.98, *p* = 0.006). In the dominant model, when the WDFY4 rs7097397 AA homozygote genotype was used as the reference group, the AG/GG genotypes were associated with a significant 1.44-fold increased susceptibility to RA (OR = 1.44, 95% CI = 1.10–1.89, *p* = 0.008) (Table 2).

### 2.3. Stratification Analyses of *ETS1* rs1128334 G/A and *WDFY4* rs7097397 A/G Polymorphisms and the Risk for RA

Stratification analyses were done to evaluate the effects of *ETS1* rs1128334 G/A and *WDFY4* rs7097397 A/G genotypes on RA risk according to age, sex, C-reactive protein (CRP) status and ACPA status (Table 3). A significantly increased risk for RA associated with the *WDFY4* rs7097397 AG genotype was evident among female patients (OR = 1.64, 95% CI = 1.17–2.28, *p* = 0.004), younger patients (OR = 1.95, 95% CI = 1.29–2.94, *p* = 0.002), CRP-negative patients (OR = 1.56, 95% CI = 1.07–2.27, *p* = 0.022) and both ACPA positive patients (OR = 1.49, 95% CI = 1.03–2.16, *p* = 0.034) and negative patients (OR = 1.49, 95% CI = 1.03–2.16, *p* = 0.034) compared with the *WDFY4* rs7097397 AA genotype. A significantly increased risk for RA associated with the *WDFY4* rs7097397 GG genotype was evident among younger patients (OR = 2.45, 95% CI = 1.35–4.43, *p* = 0.003) and CRP-negative patients (OR = 1.80, 95% CI = 1.07–3.04, *p* = 0.027) compared with the *WDFY4* rs7097397 AA genotype (Table 3).

### 2.4. Replication and Combination Study of *WDFY4* rs7097397 A/G Polymorphism and the Risk of RA

In replication cohort with 100 RA and 100 controls, no positive results were found (data not shown), which might caused by small samples. However, after we had combined discovery cohort and replication cohort, *WDFY4* rs7097397 GG homozygote genotype was used as the reference group, the GA/AA genotypes were associated with a significant increased susceptibility to RA (Table S1).

## 3. Discussion

We determined the association between the *ETS1* rs1128334 G/A and *WDFY4* rs7097397 A/G polymorphisms and the risk of RA in a Chinese population. It was the first positive finding of *WDFY4* rs7097397 A/G polymorphism and RA. We found that *WDFY4* rs7097397 A/G may be associated with the risk of RA, and that this effect was more evident in female, younger patients, CRP-negative patients and both ACPA positive and negative patients.

The function of WDFY4 is still not well characterized [6]. *WDFY4* contains WD40 domains, which covers a wide variety of functions including adaptor/regulatory modules in signal transduction, pre-mRNA processing and cytoskeleton assembly [6].

*WDFY4* rs7097397 causes non-synonymous substitution of Arg1816Gln [17]. We found that the *WDFY4* rs7097397 AG allele may increase the risk of RA, particularly in CRP-negative patients and both ACPA positive and negative patients, indicating a gene-environment interaction.

Ethnic differences may play a part in the conflicting results seen in associated studies. Our results, using the same genetic markers with subjects of the same ethnic backgrounds as those in the original studies, suggest that *WDFY4* rs7097397 A/G confers susceptibility for RA in the Chinese population.

Genetic polymorphisms often vary between ethnic groups. In the present study with 697 controls, we reported that the minor allele frequency of *ETS1* rs1128334 G/A was similar to that reported in Hong Kong and Shanghai of Chinese populations, but a little higher than in Hefei of Chinese population and Bangkok [6]. The minor allele frequency of *WDFY4* rs7097397 A/G was similar to that reported in Shanghai and Hefei of Chinese populations and Bangkok, but a little higher than in Hong Kong of Chinese population [6].

Considering mutant alleles in the control group, OR, case samples and control samples, the power of our study (α = 0.05) was 0.071 and 0.835 for *ETS1* rs1128334 G/A and *WDFY4* rs7097397 A/G respectively.

Several limitations of the present study need to be addressed. First, this was a hospital-based case–control study, so selection bias was unavoidable and the subjects were not fully representative of the general population. Second, the polymorphisms we investigated, based on their functional considerations, may not offer a comprehensive view of the genetic variability of *ETS1* and *WDFY4*, further fine mapping studies are recommend. Third, a single case–control study is not sufficient to fully interpret the relationship between the *ETS1* rs1128334 G/A and *WDFY4* rs7097397 A/G polymorphisms and susceptibility to RA because of the relatively moderate number of patients evaluated. Larger numbers of subjects are necessary to confirm our findings, especially for the results of *ETS1* rs1128334 G/A and *WDFY4* rs7097397 A/G polymorphisms and RA. Finally, we did not obtain detailed information about RA severity and the outcomes of treatment, which restricted our analyses.

## 4. Experimental Section

### 4.1. Study Populations

We obtained approval of the study protocol from the Ethics Committee of Nanjing Medical University (Nanjing, China). All patients provided written informed consent to be included in the study.

Three hundred and twenty-nine RA patients who fulfilled the criteria for RA set by the American College of Rheumatology classification in 1987 [18] were consecutively recruited from the Changzhou Second Hospital-Affiliated Hospital of Nanjing Medical University (Changzhou, China), the Changzhou First Hospital (Changzhou, China), and the Changzhou Traditional Chinese Medical Hospital (Changzhou, China), between September 2010 and October 2011. The controls were patients without RA, matched for age (±5 years) and sex, and recruited from the same institutions during the same time period; most of the controls were admitted to the hospitals for the treatment of trauma. All cases and controls were Chinese Han population. We also recruited another 100 RA cases and 100 controls without RA, matched for age (±5 years) and sex, between June 2013 and December 2013 for replication study purpose.

Each patient was interviewed by trained personnel using a pre-tested questionnaire to obtain information on demographic data and related risk factors for RA. After the interview, 2 mL of peripheral blood was collected from each subject.

Isolation of DNA and genotyping by matrix-assisted laser desorption/ionization time-of-flight mass spectrometry (MALDI-TOF MS)

Blood samples were collected using vacutainers and transferred to test tubes containing ethylenediamine tetra-acetic acid (EDTA). Genomic DNA was isolated from whole blood using the QIAamp DNA Blood Mini Kit (Qiagen, Hilden, Germany). Genotyping was done by MALDI-TOF MS using the MassARRAY system (Sequenom, San Diego, CA, USA) as previously described (Figure S1) [19]. For quality control, repeated analyses were undertaken on 10% of randomly selected samples.

### 4.2. Statistical Analyses

Differences in demographics, variables, and genotypes of the *ETS1* rs1128334 G/A and *WDFY4* rs7097397 A/G polymorphism variants were evaluated using a chi-squared test. The associations between *ETS1* rs1128334 G/A and *WDFY4* rs7097397 A/G genotypes and risk of RA were estimated by computing odds ratios (ORs) and 95% confidence intervals (CIs) using logistic regression analyses, and by using crude ORs. The Hardy–Weinberg equilibrium (HWE) was tested by a goodness-of-fit chi-squared test. All statistical analyses were done with SAS software (version 9.1.3; SAS Institute, Cary, NC, USA).

## 5. Conclusions

In conclusion, the present study provided strong evidence that *WDFY4* rs7097397 A/G functional polymorphisms may contribute to the risk of RA. However, our results were obtained from a moderate-sized sample, and therefore this is a preliminary conclusion. Validation by a larger study from a more diverse ethnic population is needed to confirm these findings.

## Supplementary Information



## Figures and Tables

**Table 1.** Patient demographics and risk factors in rheumatoid arthritis, all subjects.

Variable	Cases (*n* = 329)	Controls (*n* = 697)	*p*
Age (years)	53.64 (±15.52)	53.45 (±11.35)	0.829
Female/male	247/82	496/201	0.190
Age at onset, years, mean ± SD	44.93 (±12.55)	–	–
Disease duration, years, mean ± SD	8.76 (±9.31)	–	–
Treatment duration, years, mean ± SD	7.07 (±7.38)	–	–
RF-positive, no. (%)	266 (80.9%)	–	–
ACPA-positive, no. (%)	163 (49.5%)	–	–
CRP-positive, no. (%)	165 (50.2%)	–	–
ESR, mm/h	34.00 (±23.96)	–	–
DAS28	4.33 (±1.61)	–	–
Functional class, no. (%)		–	–
I	49 (14.9%)	–	–
II	136 (41.3%)	–	–
III	116 (35.3%)	–	–
IV	28 (8.5%)	–	–

RF: Rheumatoid factor; ACPA: Anti-cyclic citrullinated peptide; CRP: C-reactive protein; ESR: Erythrocyte sedimentation rate; DAS28: RA disease activity score.

**Table 2.** Logistic regression analysis of associations between E26 transformation-specific-1 (ETS1) rs1128334 G/A and *WDFY4* rs7097397 A/G polymorphisms and risk of rheumatoid arthritis.

Genotype	Cases (*n* = 329)		Controls (*n* = 697)		Crude OR (95% CI)	*p*	Adjusted OR (95% CI)	*p*

	n	%	n	%				
*ETS1* rs1128334 G/A								

G allele	412	64.6	880	65.4	1.00	–		
A allele	226	35.4	466	34.6	1.04 (0.85–1.26)	0.726		
GG	135	42.3	291	43.2	1.00	–	1.00	–
GA	142	44.5	298	44.3	1.03 (0.77–1.37)	0.854	1.02 (0.76–1.35)	0.921
AA	42	13.2	84	12.5	1.08 (0.71–1.65)	0.729	1.06 (0.69–1.62)	0.792
AA *vs*. GA *vs*. GG						0.939		
GA + AA	184	57.7	382	56.8	1.04 (0.79–1.36)	0.785	1.02 (0.78–1.34)	0.862
GG + GA	277	86.8	589	87.5	1.00	–	1.00	–
AA	42	13.2	84	12.5	1.06 (0.72–1.58)	0.762	1.05 (0.71–1.57)	0.807

*WDFY4* rs7097397 A/G								

A allele	404	62.9	933	67.5	1.00	–		
G allele	238	37.1	449	32.5	1.22 (1.01–1.49)	0.043		
AA	122	38.0	324	46.9	1.00	–	1.00	–
AG	160	49.8	285	41.2	1.49 (1.12–1.98)	0.006	1.50 (1.13–1.99)	0.005
GG	39	12.1	82	11.9	1.26 (0.82–1.95)	0.292	1.26 (0.82–1.95)	0.294
GG *vs*. AG *vs*. AA						0.022		
AG + GG	199	62.0	367	53.1	1.44 (1.10–1.89)	0.008	1.45 (1.10–1.90)	0.008
AA + AG	282	87.9	609	88.1	1.00	–	1.00	–
GG	39	12.1	82	11.9	1.03 (0.68–1.54)	0.897	1.02 (0.68–1.54)	0.911

The genotyping was successful in: 319 cases and 673 controls for *ETS1* rs1128334 G/A; 321 cases and 691 controls for *WDFY4* rs7097397 A/G. Adjusted for age and sex.

**Table 3.** Stratified analyses between *ETS1* rs1128334 G/A and *WDFY4* rs7097397 A/G polymorphisms and risk of rheumatoid arthritis.

Variable	*ETS1* rs1128334 G/A (Case/Control)			OR (95% CI)			*WDFY4* rs7097397 A/G (Case/Control)			OR (95% CI)		

	GG	GA	AA	GG	GA	AA	AA	AG	GG	AA	AG	GG
Gender												

Male	33/99	37/75	8/20	1.00	1.48 (0.85–2.58); *p*:0.168	1.20 (0.48–2.98); *p*:0.695	33/87	39/88	9/22	1.00	1.17 (0.67–2.03); *p*:0.579	1.08 (0.45–2.58); *p*:0.865
Female	102/192	105/223	34/64	1.00	0.89 (0.64–1.24); *p*:0.479	1.00 (0.62–1.62); *p*:1.000	89/237	121/197	30/60	1.00	1.64 (1.17–2.28); *p*:0.0037	1.33 (0.81–2.20); *p*:0.263

Age												

<55	69/143	68/156	23/34	1.00	0.90 (0.60–1.35); *p*:0.623	1.40 (0.77–2.56); *p*:0.271	55/176	79/130	26/34	1.00	1.95 (1.29–2.94); *p*:0.0016, *p*_correct_:0.0032	2.45 (1.35–4.43); *p*:0.0031, *p*_correct_:0.0062
≥55	66/148	74/142	19/50	1.00	1.17 (0.78–1.75); *p*:0.450	0.85 (0.47–1.56); *p*:0.603	67/148	81/155	13/48	1.00	1.15 (0.78–1.71); *p*:0.476	0.60 (0.30–1.18); *p*:0.137

CRP status												

Positive	67/291	72/298	20/84	1.00	1.05 (0.73–1.52); *p*:0.799	1.03 (0.59–1.80); *p*:0.906	65/324	82/285	13/82	1.00	1.43 (1.00–2.06); *p*:0.051	0.79 (0.42–1.50); *p*:0.473
Negative	68/291	70/298	22/84	1.00	1.01 (0.69–1.46); *p*:0.978	1.12 (0.65–1.92); *p*:0.678	57/324	78/285	26/82	1.00	1.56(1.07–2.27); *p*:0.0216 *p*_correct_:0.0432	1.80 (1.07–3.04); *p*:0.0273 *p*_correct_:0.0546

ACPA status												

Positive	66/291	76/298	17/84	1.00	1.12 (0.78–1.62); *p*:0.531	0.89 (0.50–1.60); *p*:0.703	61/324	80/285	19/82	1.00	1.49 (1.03–2.16); *p*:0.0340 *p*_correct_:0.0680	1.23 (0.70–2.17); *p*:0.475
Negative	69/291	66/298	25/84	1.00	0.93 (0.64–1.36); *p*:0.721	1.26 (0.75–2.11); *p*:0.390	61/324	80/285	20/82	1.00	1.49 (1.03–2.16); *p*:0.0340 *p*_correct_:0.0680	1.30 (0.74–2.27); *p*:0.365

The genotyping was successful in: 319 cases and 673 controls for *ETS1* rs1128334 G/A; 321 cases and 691 controls for *WDFY4* rs7097397 A/G; Bonferroni correction was performed to correct the *p* value (*p*_correct_).

## Abbreviations

CI: confidence interval
ETS1: E26 transformation-specific-1
WDFY4: WDFY family member 4
LD: linkage disequilibrium
OR: odds ratio
SNP: single nucleotide polymorphism
