# Supplementary material for: E26 Transformation-Specific-1 (ETS1) and WDFY Family Member 4 (WDFY4) Polymorphisms in Chinese Patients with Rheumatoid Arthritis
Source: Int J Mol Sci. 2014 Feb 17;15(2):2712–21. doi: 10.3390/ijms15022712 (PMC3958877; doi:10.3390/ijms15022712)
Supplement: Supplementary file 1 [file ijms-15-02712-s001.pdf]

Supplementary Information

Figure S1. Genotyping of *ETS1* rs1128334 G/A and *WDFY4* rs7097397 A/G by MALDI-TOF-MS.

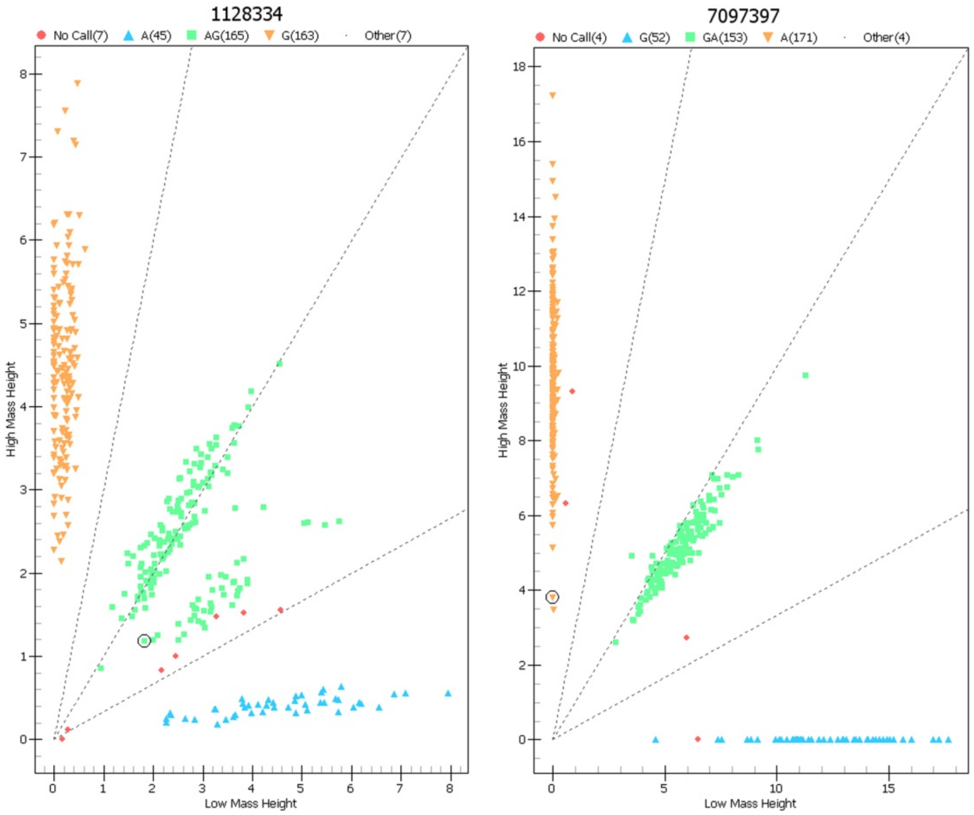

Table S1. Logistic regression analysis of associations between *ETS1* rs1128334 G/A and *WDFY4* rs7097397 A/G polymorphisms and risk of rheumatoid arthritis in 429 cases and 797 controls.

Genotype	Cases (*n* = 429)		Controls (*n* = 797)		Crude OR (95% CI)	*p*	Adjusted OR (95% CI)	*p*
	n	**%**	***n***	**%**				
WDFY4 rs7097397 A/G								
A allele	539	64.0	1065	67.3	1.00	–		
G allele	303	36.0	517	32.7	1.16 (0.97–1.38)	0.102		
AA	164	39.0	364	46.0	1.00	–	1.00	–
AG	211	50.1	337	42.6	**1.39 (1.08–1.79)**	**0.011**	**1.40 (1.08–1.80)**	**0.010**
GG	46	10.9	90	11.4	1.13 (0.76–1.69)	0.537	1.13 (0.76–1.69)	0.541
GG *vs*. AG *vs*. AA						**0.036**		
AG+GG	257	61.0	427	54.0	**1.34 (1.05–1.70)**	**0.018**	**1.34 (1.05–1.71)**	**0.018**
AA+AG	375	89.1	701	88.6	1.00	–	1.00	–
GG	46	10.9	90	11.4	0.96 (0.66–1.39)	0.814	0.95 (0.65–1.39)	0.801

The genotyping was successful in 421 cases and 791 controls for *WDFY4* rs7097397 A/G. Adjusted for age and sex.

© 2014 by the authors; licensee MDPI, Basel, Switzerland. This article is an open access article distributed under the terms and conditions of the Creative Commons Attribution license (http://creativecommons.org/licenses/by/3.0/).
